# Room-temperature unidirectional routing of valley excitons of monolayer WSe_2_ via plasmonic near-field interference in symmetric nano-slits

**DOI:** 10.1515/nanoph-2023-0368

**Published:** 2023-08-01

**Authors:** Xinglin Wen, Yunxi Zhou, Sijie Chen, Wendian Yao, Dehui Li

**Affiliations:** School of Optical and Electronic Information, Huazhong University of Science and Technology, Wuhan 430074, China; Wuhan National Laboratory for Optoelectronics, Optical Valley Laboratory, Huazhong University of Science and Technology, Wuhan 430074, China

**Keywords:** near-field interference, TMDs, valley separation, valleytronics

## Abstract

Due to the short valley polarization time, it is hardly to separate opposite valley pseudospin of transition metal dichalcogenides (TMDs) for their practical applications in valleytronics. Coupling TMDs to unidirectional surface plasmon polariton (SPP) can overcome this obstacle. However, it is required to break the symmetry to induce the asymmetric coupling between valley exciton dipole and SPP to route valley exciton in previously proposed strategies. Herein, by utilizing a new mechanism that near-field interference can create directional SPP in symmetric nanostructures, we realize directional routing of valley exciton emission of monolayer WSe_2_ at room temperature with a symmetric nano-slits array. The near-field interference enabled directional SPP in our device not only render the exciton diffusion length increase from 0.9 to 3.0 μm, but also lead to a valley exciton separation length of 0.7 μm with degree of valley polarization up to 22 %. This valley excitons separation is attributed to the non-flat WSe_2_ in the nano-slits region, which makes the exciton dipoles present in-plane and out-of-plane simultaneously. Our work provides a convenient and promising strategy towards room temperature on-chip integrated valleytronic devices.

## 1 Introduction

Valley is referred to energy extrema at either conduction/valence band occurring at high-symmetry points in the lattice. In addition to charge and spin, valley can also serve as an alternative degree of freedom to encode and store information, which is known as “valleytronics” [1–5]. Although the idea of using valley as information carrier was proposed long time ago [6, 7], the development of this field was constrained until the recent emergence of transition metal dichalcogenides (TMDs).

To encode information with valley pseudospin in TMDs, the current research focus lies in comprehending how to manipulate free carriers (electrons and holes) or composite quasiparticles (excitons) residing in opposite valleys. It is well known that excitons in TMDs possess binary valley pseudospins which can be excited and detected with circularly polarized light, namely, right circularly polarized (σ+) and left circularly polarized (σ–) excitation only induce corresponding σ+ and σ– photoluminescence (PL) [2, 4, 5]. Nevertheless, the relatively short valley depolarization time renders that valley-polarized PL can be only observed at low temperature and the degree of valley polarization *ρ* = (*I*_σ+_ − *I*_σ−_)/(*I*_σ+_ + *I*_σ−_) (DVP) is far away from unity. Although various strategies have been proposed to increase the operation temperature and enhance DVP including applying magnetic field, electric controlling and chalcogenide alloying [8–11], it is still challenging to realize high DVP at room temperature.

Recently, it has been demonstrated that plasmonic nanostructures or metasurface could be used to couple with valley excitons to overcome the obstacle of short valley depolarization time [12–17]. For instance, due to the propagation direction of surface plasmon polariton (SPP) and circular polarization of incidence light are locked in a metasurface consisting of asymmetric grooves, the valley polarized PL of monolayer MoS_2_ could be spatially separated at room temperature [14]. In addition, valley-selective unidirectional exciton emission was also realized in some other plasmonic and photonic platforms [18–21]. Nevertheless, so far, the spatial separation of valley exciton emission was induced by breaking the symmetry of structures. The asymmetric structures would render the fabrication much complicated. Therefore, it is of great significance to realize robust valley exciton routing in a symmetric plasmonic or metasurface system to make it more suitable for practical applications.

It has been demonstrated that counterintuitive unidirectional SPP launching could be achieved by coupling a circularly polarized dipole with a spatially symmetric gold nano-slit [22]. The unidirectionality of SPP originates from the near-field interference between transverse and longitudinal components of evanescent field [23–25], which provides a robust approach to manipulate the SPP direction under σ+ and σ– excitation. It is worthy to be noted that unidirectional SPP in a symmetric nano-slit requires dipole has an out-of-plane component as discussed in literature [22]. Herein, by using near-field interference, we realize room-temperature routing of valley exciton emission in monolayer (ML) WSe_2_/symmetric nano-slit array coupled system. We found the exciton diffusion length increased from 0.9 to 3.0 μm under the assistance of SPP near fields. More importantly, directional routing of valley exciton emission was observed at room temperature with DVP between –15–22 % and propagation length was around 0.7 μm. Simulations indicate that the valley exciton separation in direction perpendicular to the slit can be attributed to the near-field interference.

## 2 Results and discussions

**Figure 1:**
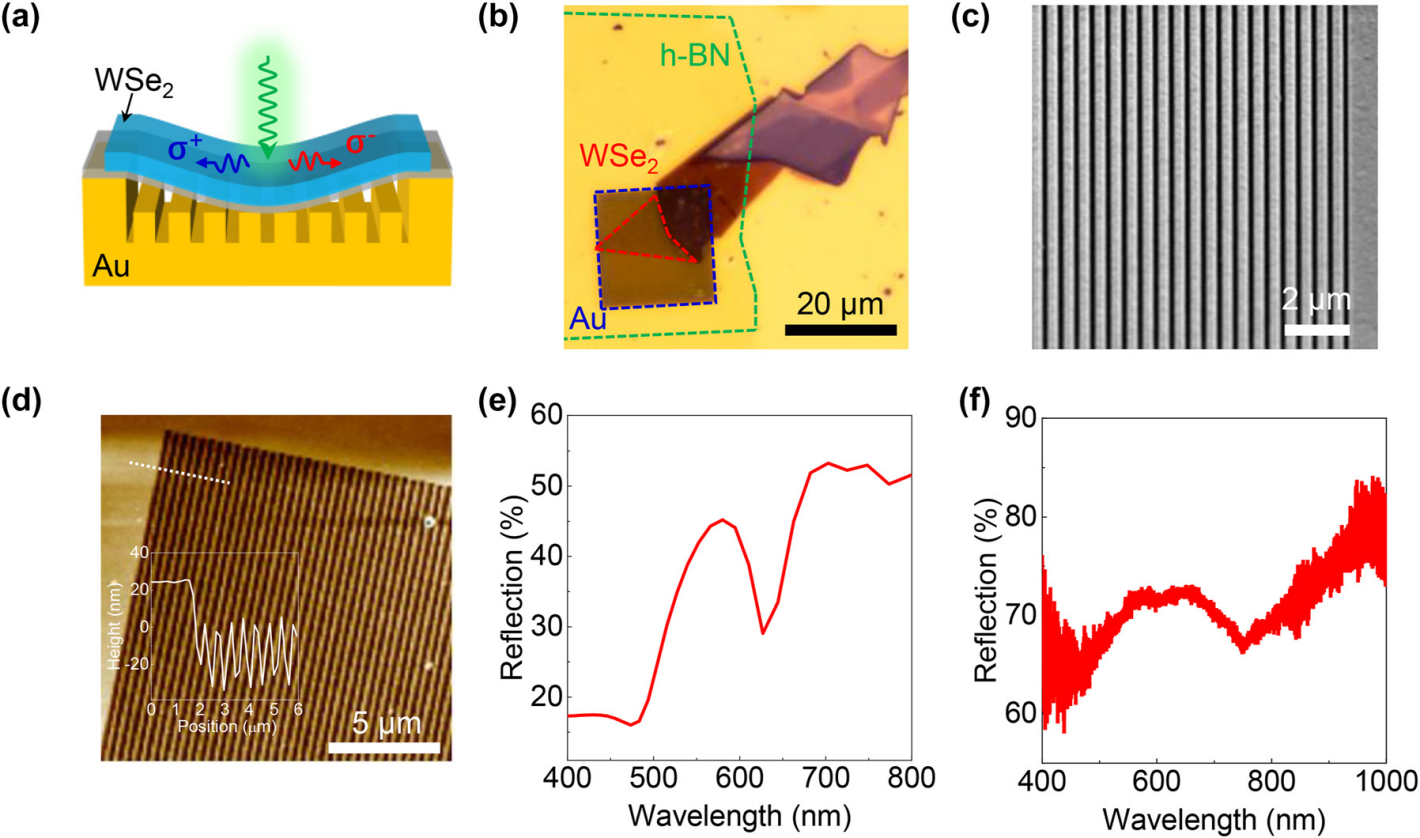
Device design and fabrication. (a) Schematic of device structure and working principle. Valley exciton dipoles of curved WSe_2_ have both in-plane and out-of-plane components, which excite the nano-slit to induce unidirectional propagation via near-field interference. (b) Optical microscopy image of as-fabricated sample. Au nano-slit array, ML WSe_2_ and h-BN spacer are indicated by blue, red and green dashed line, respectively. (c) SEM image of Au slits array. (d) AFM image of Au slits array. The insert figure is the height profile along the dashed line. (e) and (f) Are simulated and measured reflection.

The device configuration is schematically illustrated in Figure 1a. As discussed above, unidirectional SPP in a symmetric nano-slit requires that the circularly polarized dipole is tilted respect to the horizontal plane [22]. In order to realize unidirectional valley emission, we tilt the exciton dipole orientation of ML WSe_2_ by introducing a curve as displayed in Figure 1a. More details regarding the working principle will be discussed later in the simulation results. As displayed in Figure 1b, Au nano-slit array with area of 20 × 20 μm^2^ (blue dashed line) was fabricated via focus ion beam (FIB) lithography. Subsequently, ML WSe_2_ (red dashed line) was transferred onto slit array with h-BN layer (green dashed line) as spacer to block the charge transfer. Scanning electron microscopy (SEM) image in Figure 1c indicates the uniformity of the as-fabricated nano-slit array. Atomic force microscopy (AFM) image and the corresponding height profile in Figure 1d show that depth of slit is around 30 nm. The inserted height profile in Figure 1d also shows that the whole slit array area is thinned around 20 nm because of electron exposure, which is consistent with our device design in Figure 1a that WSe_2_ is curved. Figure 1e is the simulation of reflection of slit array with width of 500 nm, periodicity of 1 μm and depth of 30 nm. Reflection is simulated via finite-difference time-domain (FDTD) solutions (Lumerical Solutions). In the simulations, a plane wave source with linear polarization perpendicular to the slits is selected as the incident field and perfectly matched layers (PML) are defined as the boundary condition. A monitor is placed on the incidence side to record the reflection signal. The dielectric constant of gold is adopted from the value measured by Johnson and Christy [26]. The dip at 630 nm is attributed to the excitation of gap surface plasmon of the metallic slits with the electromagnetic field confined in the groove [27, 28]. The measured reflection spectrum in Figure 1f presents a dip at 752 nm and this deviation may be ascribed to non-ideal FIB lithography. However, the similar shape of the reflection curve indicates that the dip indeed come from the plasmonic resonance. Though plasmon resonance will red-shift slightly after putting h-BN and WSe_2_ due to the refractive index change, the surface plasmon resonance of 752 nm has broad overlap with the exciton emission of ML WSe_2_ at room temperature.

**Figure 2:**
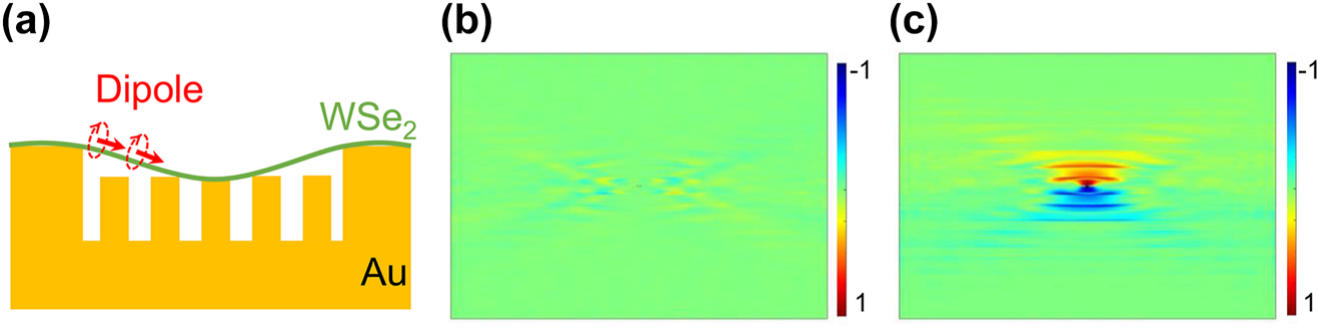
Simulations of electric fields. (a) Schematic of the simulation for a tilted dipole. (b) and (c) Are simulated contrast image of circularly polarized near field (*ρ* = (*I*_R_ − *I*_L_)/(*I*_R_ + *I*_L_)) under normal and tilted dipole (10°), respectively.

To further illustrate the working principle of our device, we simulated degree contrast of electric field *ρ* = (*I*_R_ − *I*_L_)/(*I*_R_ + *I*_L_) under excitation of a valley polarized dipole. Because valley polarized emission of ML TMDs can be represented by opposite circularly polarized dipole [14, 15, 19], a right or left circularly polarized dipole was placed on top of slit array to simulate PL emission from +K and –K valley of ML WSe_2_, respectively. The mechanism of near-field induced unidirectional SPP in a symmetric nano-slit is attributed to the interference between out-of-plane dipole driven transverse evanescent field and in-plane dipole driven longitudinal evanescent field. Consequently, destructive and constructive interference in two opposite directions lead to the unidirectional SPP. In other words, dipole must present in-pane and out-of-plane components simultaneously to achieve unidirectional SPP. Considering the exciton is confined in-plane for ML WSe_2_, it is supposed that valley separation will be absent if WSe_2_ is flat. Indeed, the simulated electric field distribution of in-plane circularly polarized dipole indicates there is no unidirectional SPP (Figure 2b). In the contrast, the non-flat WSe_2_ renders that circularly polarized dipole has an additional out-of-plane component as schematically illustrated in Figure 2a. Notable directional SPP propagation is resolved if dipole has a typical title angle in Figure 2c. To sum up, valley exciton dipoles of our curved WSe_2_ have both in-plane and out-of-plane component, which will excite the symmetric nano-slit to induce unidirectional valley emission.

PL spectra were measured under a 633 nm laser excitation at room temperature (300 K) to study the valley exciton emission. As shown in Figure S1a (Supporting Information), valley polarization was not preserved at room temperature under circularly polarized light excitation, which is caused by the dominant inter-valley scattering at room temperature. Furthermore, PL was also measured under orthogonal linear excitation and the same results were obtained with vanished valley polarization (Figure S1b). PL of ML WSe_2_ on Au film and slit array were measured (Figure S2), respectively. A PL redshift (∼8 nm) was observed for ML WSe_2_ on slit array, which is attributed to the strain effect [29] originated from the reduced thickness in FIB process as shown in Figure 1d. The strain effect verifies that the ML WSe_2_ is curved.

Although the total PL collected by objective is not valley polarized, the spatial distribution of DVP is probably non-zero because of the SPP-exciton coupling. Therefore, spatially resolved PL image was recorded with micro-Raman spectrometer (Figure S3). PL image was recorded by a charge-coupled-device (CCD) and has resolution of 0.2 μm under 100× objective, making it capable to measure spatially distributed PL in vicinity of laser spot. Actually, this kind of setup was frequently used to trace excitons’ dynamical behavior such as exciton diffusion and valley polarization distribution [30, 31]. Both laser spots (633 nm) illuminated on Au film and slit array were measured as references (Figure S4). Full-width-at-half-maximum (FWHM) of laser spots (Gaussian fitting) on film and slit array are 0.50 and 0.49 μm, respectively, indicating the scattering effect on excitation laser is negligible.

**Figure 3:**
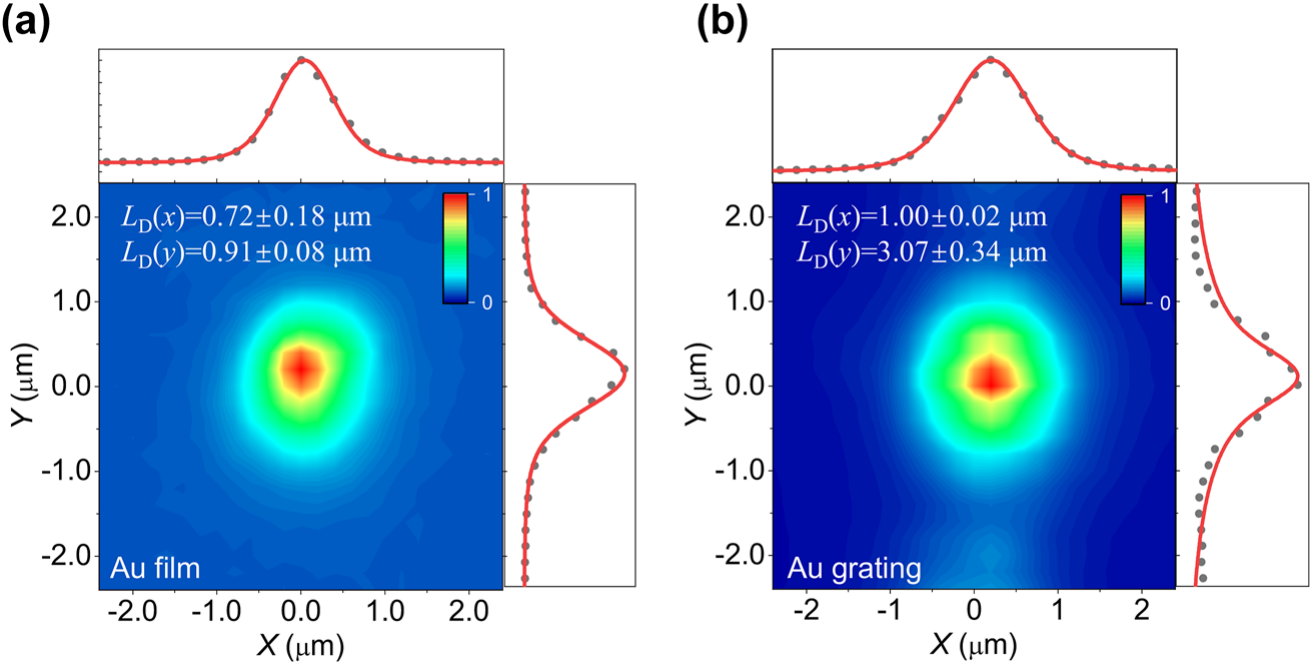
Exciton diffusion. (a) and (b) Are PL image of ML on Au film and Au nano-slit array respectively. The PL intensity along *x* and *y* direction are shown in black dots at top and right panels. Red lines is the fitting curve and the fitted exciton diffusion length along *x* and *y* direction are also labelled in each picture.

Figure 3a and b are normalized PL image of ML WSe_2_ placed on Au film and Au slits array, respectively. PL intensity along parallel (*x*−) and perpendicular (*y*−) direction are extracted as black dots in top and right panels. The orientation of slit is along parallel direction (*x*−) in Figure 3b. To gain understanding how slit array influence exciton transport, the exciton diffusion length was estimated. In steady state conditions, excitons concentration (PL intensity) can be described by the convolution between laser’s Gaussian function and Bessel function of the second kind *K*_0_ [32].

$$I=d+A\overset{+\infty }{\underset{-\infty }{\int }}K_{0}\left(r^{\prime }/L_{D}\right)e^{-\frac{{\left(r-x_{0}-r^{\prime }\right)}^{2}}{w^{2}}}dr^{\prime }$$

where *L*_*D*_, *w*, *x*_0_, *A*, *d* is diffusion length, radius of laser spot, excitation position, normalized coefficient and baseline, respectively. The fitted diffusion length along parallel (*L*_*D*_(*x*)) and perpendicular (*L*_*D*_(*y*)) directions are presented in Figure 3a and b. The diffusion length of ML WSe_2_ on Au film is almost closed (0.7∼0.9 μm) in both parallel and perpendicular directions, indicating Au film has negligible influence on exciton diffusion. This is because guided SPP cannot be excited directly on bare Au film without momentum match. Diffusion length in parallel direction (*x*−) for sample on slit is also closed to Au film scenarios, whereas diffusion length along perpendicular direction (*y*−) is around 3 μm, longer than Au film cases. The longer diffusion length is attributed to the coupling between exciton and guided SPP propagating along perpendicular direction. The guided SPP of slit array can be routinely excited with linear polarized incidence. Consequently, the exciton transport is modified by the near-filed non-radiative energy transfer *via* exciton-SPP-exciton conversion process as discussed in previous reports [14].

**Figure 4:**
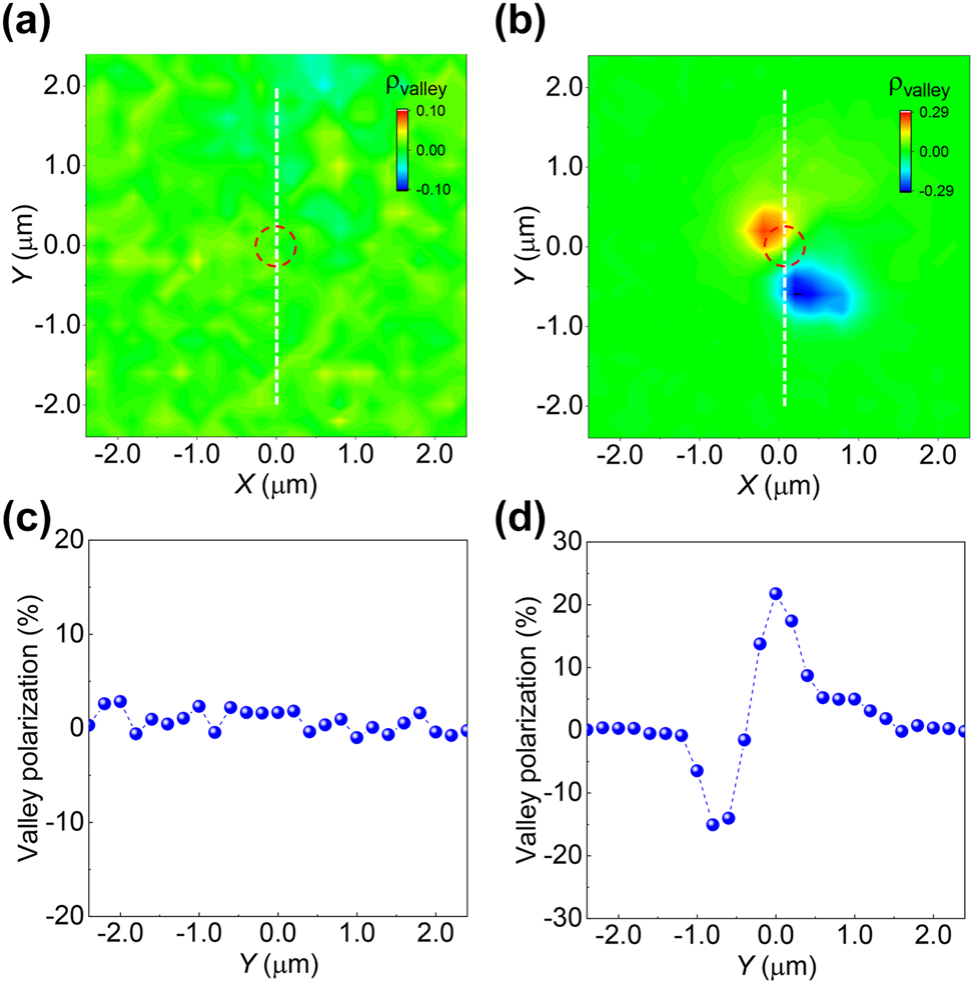
Valley excitons seperation. (a) Valley polarization (*ρ* = (*I*_σ+_ − *I*_σ−_)/(*I*_σ+_ + *I*_σ−_)) image of ML WSe_2_ on Au film. (b) Valley polarization images of ML WSe_2_ on Au slits under perpendicular (*y*) linear polarized laser excitation. Red dashed circles indicate the laser spots and the orientation of slits is along *x* direction. (c) and (d) Are the extracted valley polarization curves along the white dashed lines in (a) and (b),respectively.

To investigate the characteristics of valley exciton propagation, spatially- and circular polarization- resolved real-space PL images were recorded by sequently inserting a polarizer, *λ*/2 and *λ*/4 plate in front of CCD slit. The DVP distribution image was produced by formula *ρ* = (*I*_σ+_ − *I*_σ−_)/(*I*_σ+_ + *I*_σ−_) at each image pixel. DVP distribution image was firstly measured for ML WSe_2_ on SiO_2_/Si for reference (Figure S5) and the DVP was near zero at every position as expected, verifying the setup was reliable. For ML WSe_2_ on Au film in Figure 4a under linear excitation, valley polarization is not observed neither. This is because SPP is not excited on surface of Au film as discussed above. In contrast, prominent DVP distribution was observed for ML WSe_2_ on slit array under linear excitation perpendicular to slit as shown in Figure 4b. Notably, excitons from +K (σ+) and –K (σ–) valley prefer to diffuse toward opposite direction. This is because that it is the excitons that excite the SPP but not the laser excitation within excitons-SPP-excitons energy conversion process [14]. The linearly polarized laser excites both +K and −K valley exciton, namely, a left and right circularly polarized dipole. These dipoles render SPP propagate in the direction perpendicular to the slits as shown in Figure 2, which is consistent with our observation. In addition, we also measured the DVP distribution by rotating the sample by 90° and the valley excitons were also separated along the direction perpendicular to slits (Figure S6). Likewise, the DVP is almost zero at center (near laser spot) because σ+ and σ– PL coincide with each other within the laser spots, which is consistent with the previous report of exciton Hall effect [30]. Figure 4d is the extracted DVP along the dashed white line in Figure 4b, and the opposite sign of DVP is clearly visualized. The distance between the separated peaks (σ+ and σ–) is around 0.7 μm. The absolute σ+ and σ– PL is shown in Figure S7 and DVP varies from −15 to 22 % at different positions. The separation length is shorter than previous report (1.2 μm) based on asymmetric silver groove [14]. This is because SPP propagation length of evaporated Au film we used is shorter than the single-crystalline silver film.

Simulation in Figure 2c shows that valley excitons should be separated perfectly along the vertical direction, while the experimental result of the valley separation exhibits a small rotation angle respect to the vertical axis (Figure 4b). We interpret that this deviation is most probably attributed to the rotation of the sample during the measurement. In the measurement, we identified the orientation of the nano-slits by looking at the square of the array (Figure 1b) under microscope. Thus, we could not see the individual nano-slit under the optical microscope. Consequently, the nano-slits may not be aligned horizontal perfectly. However, the valley separation in experiments follows the directional SPP propagation trend in simulation. To sum up, the directional valley exciton emission can be explained by following steps. The linear excitation laser could induce linear PL emission at room temperature which could be decomposed in to σ+ and σ– emission. Afterwards, σ+ and σ– emission induce the near-field interference and propagate to opposite direction as discussed above, leading to the separation of valley exciton emission.

## 3 Conclusions

In contrast to the conventional strategy of breaking symmetry, we demonstrated a new working principle to route valley exciton with symmetric structure. Symmetric Au nano-slits array was proposed to route the valley exciton emission of ML WSe_2_ at room temperature. Coupled with Au nano-slits, exciton diffusion length of ML WSe_2_ was increased from 0.9 to 3.0 μm because of the SPP-exciton interaction and the σ+ and σ– emission was demonstrated to propagate to opposite direction, leading to the valley exciton separation in real space. A separation length of 0.7 μm was obtained with DVP up to 22 %. The valley exciton separation is originated from the unidirectional SPP propagation induced by the near-field interference and could be achieved under perpendicular linear excitations. Our results provide a new mechanism to route valley excitons with symmetric nano-slits, which can reduce the difficulties of device fabrication. The nano-slits enabled valley exciton routing is of great importance towards integrated excitonic circuits in the future.

## 4 Methods

### 4.1 Sample preparation

80 nm Au film with 5 nm Cr adhesion layer was firstly deposited on Si substrate *via* thermal evaporation. Subsequently, slits were created on Au film directly with focus ion beam milling method (FEI Quanta 3D FEG FIB). ML WSe_2_ and h-BN flake were mechanically exfoliated from commercial crystals (HQ graphene) and then transferred on slit array with precise alignment under microscope.

### 4.2 Optical measurement

Reflectance and PL were measured on a Raman spectrometer (Horiba, iHR 550) equipped with a liquid nitrogen cooled CCD. The signal was collected *via* back scattering mode with a 100× objective. The PL image was recorded with CCD directly by setting the grating in zero order. Left and right circularly polarized PL was collected by rotating the *λ*/4 plate. The excitation laser was 633 nm and all the measurements were performed at room temperature.

## Supplementary Material

Supplementary Material Details
