# Supplementary material for: Room-temperature unidirectional routing of valley excitons of monolayer WSe2 via plasmonic near-field interference in symmetric nano-slits
Source: Nanophotonics. 2023 Aug 1;12(17):3529–34. doi: 10.1515/nanoph-2023-0368 (PMC11501598; doi:10.1515/nanoph-2023-0368)
Supplement: Supplementary file 1 — Supplementary Material Details [file j_nanoph-2023-0368_suppl_001.pdf]

Supporting Information

Room-temperature unidirectional routing of valley excitons of monolayer WSe_2 *via* plasmonic near-field interference in symmetric nano-slits

Xinglin Wen[1,2,], Yunxi Zhou[1], Sijie Chen[1], Wendian Yao[1], Dehui Li[1,2,*]*

[1]School of Optical and Electronic Information, Huazhong University of Science and Technology, Wuhan 430074, China

[2]Wuhan National Laboratory for Optoelectronics, Optical Valley Laboratory, Huazhong University of Science and Technology, Wuhan 430074, China

*Correspondence to: Email: wenxl@hust.edu.cn and dehuili@hust.edu.cn

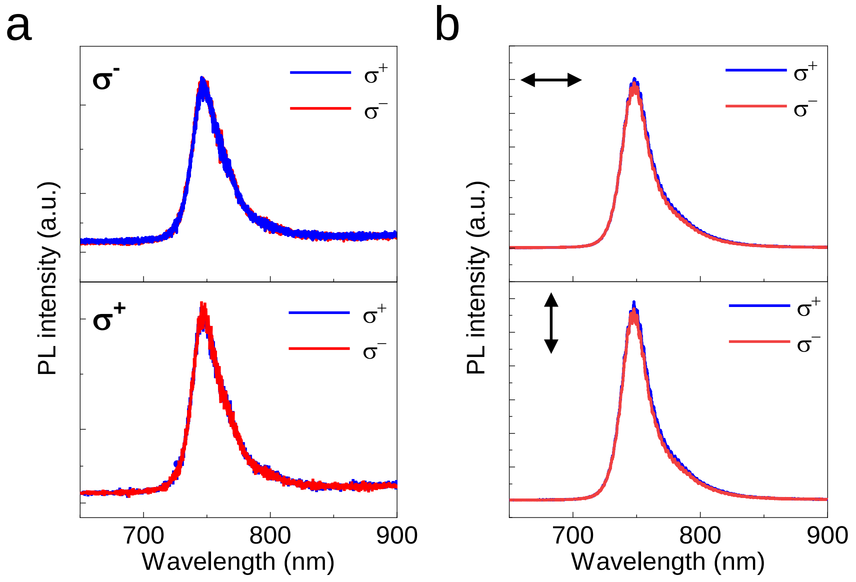

Figure S1. (a) Valley polarized PL under left-handed (upper) and right-handed (bottom) circular polarized excitation. (b) Valley polarized PL of ML WSe_2 under parallel (upper) and perpendicular (bottom) linear excitation. All the measurements were performed at room temperature under a 633 nm laser excitation.

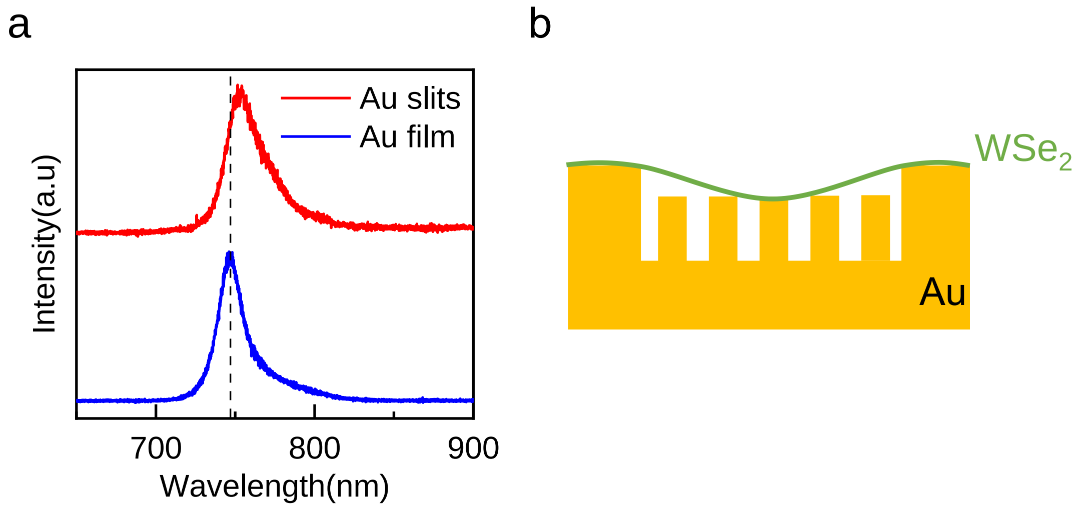

Figure S2. (a) PL of ML WSe_2 on Au film and slit array; (b) Schematic of the strain effect.

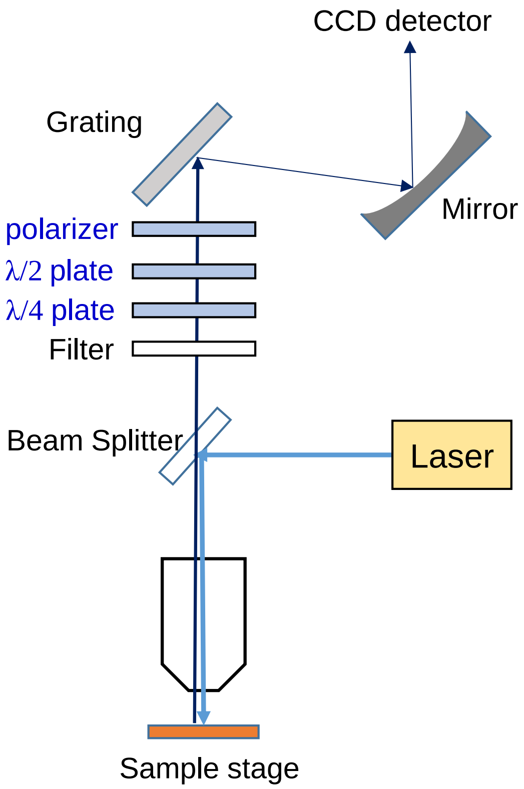

Figure S3 Schematic of the setup measuring the image of valley polarization.

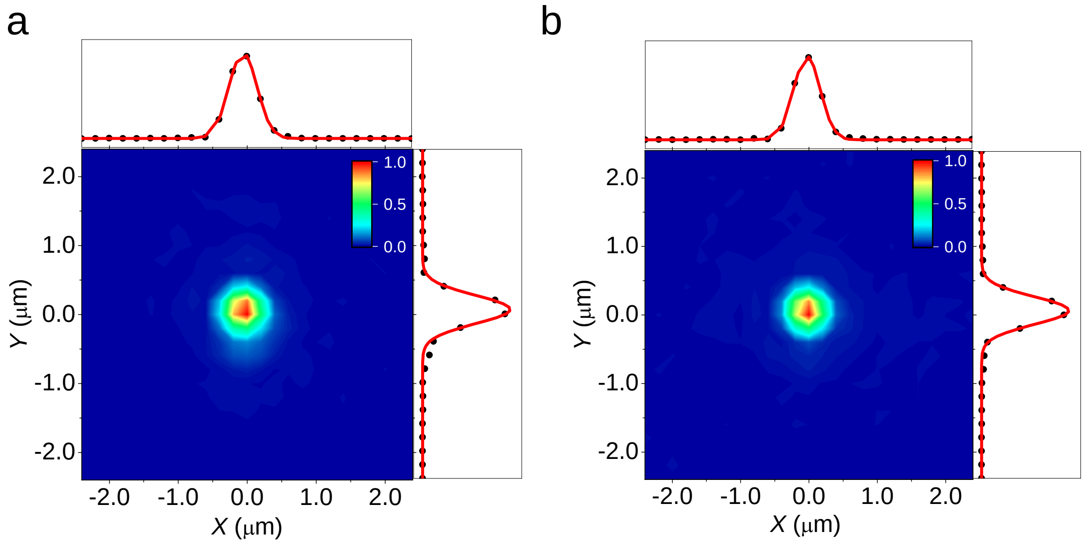

Figure S4. Image of laser spots on (a) Au film and (b) Au slit array. The intensity and Gaussian fitting along *x* and *y* direction are shown at top and right panels.

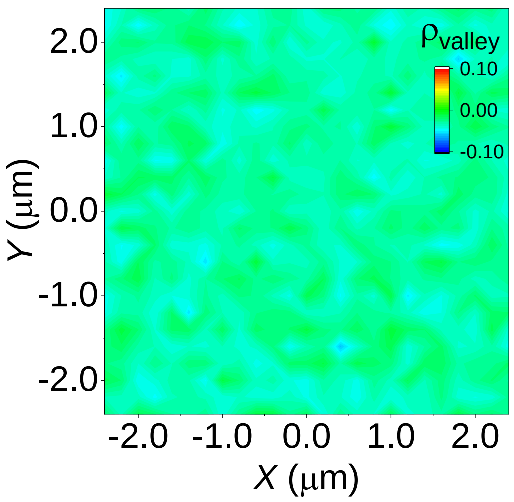

Figure S5. Valley polarization image of ML WSe_2 on SiO_2/Si substrate. Valley contrast was not observed.

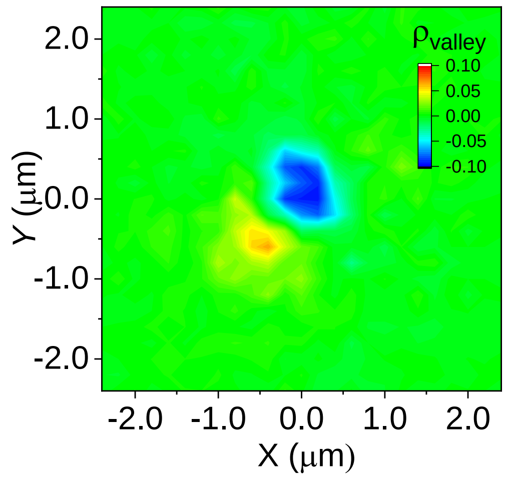

Figure S6. DVP distribution by rotating the slits by 90°, slits are along vertical direction after rotation.

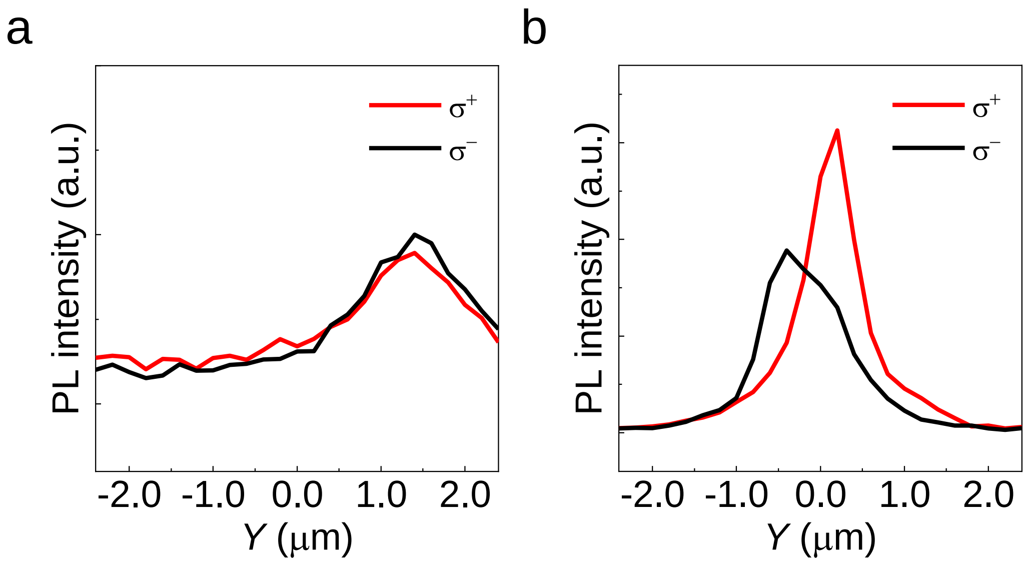

Figure S7. (a) Extracted σ+ and σ– PL of ML WSe_2 on Au film. (b) is extracted σ+ and σ– PL of ML WSe_2 on Au slits under perpendicular linear polarized laser excitation.
